# Insufficient iodine intake in pregnant women in different regions of the world: a systematic review

**DOI:** 10.20945/2359-3997000000202

**Published:** 2020-03-04

DOI: 10.20945/2359-3997000000151

Arch Endocrinol Metab. 2019;63(3):306-11

Where you read:

**Table 1:** Description of the studies selected for systematic review

Authors / Year	Site	Study Design	Recruitment	Sample size (n)	Trimester of pregnancy	Median Urinary Iodine Concentration (UIC) (μg/L)	Median UIC Classification	Prevalence of Iodine Deficiency	Quality of the Studies Included
Amouzegar and Azizi (2013) (19)	Turkey	Cross-sectional	Pregnant women referred to the mother and child health care clinics of two maternity hospitals of Tehran	36	First trimester (< 15 weeks)	138.4 (24.1 – 404)	Insufficient	34.0%	16 points

Should read:

**Table 1:** Description of the studies selected for systematic review

Authors / Year	Site	Study Design	Recruitment	Sample size (n)	Trimester of pregnancy	Median Urinary Iodine Concentration (UIC) (μg/L)	Median UIC Classification	Prevalence of Iodine Deficiency	Quality of the Studies Included
Amouzegar and Azizi (2013) (19)	**Iran**	Cross-sectional	Pregnant women referred to the mother and child health care clinics of two maternity hospitals of Tehran	36	First trimester (< 15 weeks)	138.4 (24.1 – 404)	Insufficient	34.0%	16 points

